# Real-Time Elicitation of Moral Emotions Using a Prejudice Paradigm

**DOI:** 10.3389/fpsyg.2012.00275

**Published:** 2012-08-06

**Authors:** Melike M. Fourie, Nadine Kilchenmann, Susan Malcolm-Smith, Kevin G. F. Thomas

**Affiliations:** ^1^ACSENT Laboratory, Department of Psychology, University of Cape TownCape Town, South Africa

**Keywords:** emotion elicitation, moral emotions, guilt, implicit association test, behavioral inhibition

## Abstract

Moral emotions are critically important in guiding appropriate social conduct. Empirical investigation of these emotions remains a challenge, however, because of the difficulty in eliciting them reliably in controlled settings. Here we describe a novel prejudice paradigm that aimed to elicit both negatively and positively valenced moral emotions in real-time. Low-prejudice females (*N* = 46) who met highly specific demographic and personality-based screening criteria completed a series of Implicit Association Tests (IATs). Feedback following these IATs was pre-programmed to either endorse participants’ non-prejudiced self-standards (positive condition), or to contradict their self-standards (negative condition), in response to sensitive social topics. Neutral condition IATs reflected participants’ attitudes toward non-sensitive social topics. Results demonstrated that the IATs were successful in eliciting moral-positive emotions (satisfaction and pride) and moral-negative emotions (primarily guilt). In addition, participants high in self-reported punishment sensitivity, as assessed by the Behavioral Inhibition System (BIS) scale, reported greater guilt.

Over the past 20 years, basic emotions such as fear, happiness, and anger have been the main focus of psychological and neuroscientific research into affective processing (Cacioppo et al., 2000; LeDoux, 2000; Rainville et al., 2006). Consequently, we have a better understanding of these emotions’ functional organization, behavioral motivation, and neuroanatomical correlates. In contrast, empirical studies on the more complex moral emotions that arise in social contexts, such as guilt, embarrassment, and compassion, have become prominent only recently (Haidt, 2003; Moll et al., 2008). Although interest in these social emotions is increasing rapidly, the complexities of conducting research in this area are considerable.

Moral emotions are powerful motivational forces that help us distinguish between right and wrong. Failure in the appropriate production of these emotions has been associated with several neurologic and psychiatric disorders, and may lead to amoral or socially inappropriate behavior (Muller et al., 2003; Pardini et al., 2003; Sturm et al., 2008; Blair, 2010). Guilt, shame, embarrassment, and pride are moral emotions that belong to a small family of self-conscious emotions. They are called “self-conscious” because the self is the object of evaluation, and because they are evoked by either implicit or explicit self-reflection (Tangney et al., 2007). As self-reflection occurs, these emotions provide immediate feedback on behavior and may take on a punishing or reinforcing role in terms of the event’s moral acceptability (Tracy and Robins, 2004).

A new wave of emotion research is underscoring the importance of studying emotions in real emotion-evoking situations, in scenarios that are personally relevant to participants and where emotions are experienced in real-time (e.g., Williams and DeSteno, 2008; Fourie et al., 2011). This approach is believed to be more ecologically valid than elicitation methods employing hypothetical or remembered scenarios, which may rely more on intellectual contemplation and may differ notably from emotional reactions to actual events (Herrald and Tomaka, 2002; Wilson and Gilbert, 2005; Harmon-Jones et al., 2007).

The task of eliciting authentic emotional responses in the laboratory is, however, particularly problematic for researchers studying the self-conscious emotions (Tracy and Robins, 2004). Whereas basic emotions can, for example, be elicited reliably through use of specific affectively laden media, the elicitors of self-conscious emotions can be viewed as internal cognitive events (Lewis, 2000). Specific self-conscious emotions thus do not appear to be distinguished purely by the nature of external stimuli, or by the types of situations that elicit them; rather, it is the manner in which individuals perceive the situation that defines the emotion. Furthermore, self-conscious emotions are founded in social relationships, and have been argued to arise expressly from concerns about *others*’ evaluation of the self, whether they are real or imagined (Leary, 2004). These emotions also appear to be strongly influenced by the cultural context and personal beliefs of participants (Bierbrauer, 1992; Haidt, 2003). Finally, deception is often necessary to evoke intense self-conscious emotions, which in turn raises ethical concerns (Harmon-Jones et al., 2007). These factors thus make the laboratory-based study of self-conscious emotions particularly tricky.

Moral emotion elicitation paradigms used in previous studies include: (i) script-driven imagery, where participants are required to recall and relive a past emotive experience (Wagner et al., 2011), (ii) picture or film stimuli that are used to elicit the emotions (Moll et al., 2002), (iii) vignettes where participants have to imagine their reactions to hypothetical moral scenarios (Finger et al., 2006; Kédia et al., 2008), and (iv) experimental contexts, by far in the minority, where participants are placed in real, emotion-evoking situations that pertain to their actual behavior (Herrald and Tomaka, 2002). The problem with re-living a previous emotional experience is that it is different phenomenologically from the original emotional encounter and constitutes, at best, a weaker form of the original emotion (Shin et al., 2000). Likewise, emotion induction through visual stimuli, such as emotion-laden films, is limited by several factors (Rottenberg et al., 2007). For example, a major problem with this approach is that films are high in cognitive demand and attentional capture, which will impact on the overall emotional response (Levenson, 2003). Finally, reading short emotive vignettes may be associated more with making emotional judgments in line with task demands, rather than with experiencing real affect (Levenson, 2003). In this regard, Casebeer (2003) pointed out that moral cognition is genuine – our moral equipment (including the moral emotions) evolved to effectively coordinate *actual* behavior, not hypothetical scenarios.

With this discussion in mind, we developed and tested a prejudice paradigm to elicit moral emotions in real-time. Our aim was to contribute to the study of moral emotions by devising an emotion elicitation paradigm of high ecological validity that would elicit real affective states relevant to the participant. The paradigm relied on a central finding in social psychological research into prejudiced attitudes: frequently, people who claim to hold egalitarian attitudes toward certain social groups show prejudiced tendencies on implicit measures of prejudice (Banaji and Greenwald, 1995; Phelps et al., 2000; Devine et al., 2002). These contradictory responses appear to emerge because conscious decisions to renounce prejudice against those who are different (e.g., in their ethnicity, gender, or sexual preference) do not necessarily rid us of prejudiced responses (Allport, 1954; Devine, 1989). Of importance to the present study, however, is that discrepancies between personal standards and actual responses (of prejudice) are associated with distinct affective consequences. Previous research has demonstrated that low-prejudice individuals typically experience guilt when they transgress their well-internalized moral standards (Monteith, 1993; Monteith et al., 2002; Amodio et al., 2007), whereas pride ensues when our actions conform to or uphold a personal moral value (Moll et al., 2008; Zahn et al., 2009).

In our paradigm, we presented a carefully selected sample of low-prejudice individuals with feedback of either high or non-existent prejudice following several Implicit Association Tests (IATs). The IAT is a reaction-time task designed to measure implicit influences on behavior by assessing the strengths of automatic associations between mental representations of objects (Greenwald et al., 1998). Instead of calculating actual IAT results, however, all IAT feedback in our task was pre-programmed to ensure the elicitation of moral-negative and moral-positive affect, respectively. More specifically, participants were led to think that they had transgressed their personal moral values (guilt induction), or that they had responded in accordance with their personal moral values (pride induction).

Although previous research has demonstrated that implicit racial biases on the IAT may lead to feelings of guilt (Monteith et al., 2001, 2002), the IAT paradigm we developed here contributes uniquely to the study of moral emotion elicitation. In particular, in a departure from previous IAT studies, we did not rely on participants’ implicit biases toward a specific social group to determine the IAT outcome; rather, we fitted all IATs with carefully scripted, pre-programmed feedback. This strategy sought to ensure the elicitation of target emotional states within each individual, rather than only in a subset who performed according to expectations. In order to make the feedback more plausible (and hence secure the success of the manipulation), however, we screened and selected all volunteers for participation based on several demographic and personality-based criteria. The current study thus describes a paradigm of high practical value to elicit not only moral-negative, but also moral-positive, emotions.

## Materials and Methods

### Design and participants

The initial pool of participants (*N* = 280) was drawn from a population of female undergraduate students between the ages of 18 and 25. These individuals were recruited through web-based notification and advertisements on noticeboards, and completed a 30-min web-based screening survey (Stage I). Based on responses to that survey, we selected a smaller sample (*N* = 48) to participate in the laboratory-based emotion elicitation experiment (Stage II). We used a female-only sample to reduce possible sex-related variation in emotional experience (Shields, 1991).

Participants received course credit in exchange for participation. Study procedures were approved by the Research Ethics Committees of the UCT Department of Psychology and the UCT Faculty of Health Sciences. After exclusion of two participants based on high scores (>20) on the Beck Depression Inventory – Second Edition (BDI-II; Beck et al., 1996), the final sample consisted of 46 females (age: *M* = 20.20; *SD* = 2.77).

### Procedure and materials

We required participants in the laboratory elicitation paradigm to be screened and selected carefully, and so the study was, as mentioned above, divided into two stages. Stage I, the screening and selection stage, was aimed at identifying individuals who met eligibility criteria to participate in Stage II, the actual experiment, during which they would perform various IATs and receive bogus feedback.

#### Stage I: screening and selection

Participants completed an online survey consisting of several measures. The survey began with a *demographic section* that asked for information about sex, age, race, religion, sexuality, and home language. To secure the success of the experimental manipulation, demographic inclusion criteria included being female, between the ages of 18 and 25, White, heterosexual, non-Jewish, and English-speaking.

The second section of the survey required participants to complete the Internal and External Motivation to Respond without Prejudice scales (IMS/EMS; Plant and Devine, 1998), which were modified for our purposes to have Black as well as homosexual people as target social categories. We used this measure to identify individuals who were internally motivated to respond without prejudice toward those targets (i.e., those with high IMS scores). High IMS individuals’ non-prejudiced values are thought to be integrated into their self-concepts, making them more prone to experience specific feelings of guilt following a personal moral transgression (Higgins, 1987; Plant and Devine, 1998). We also sought to identify individuals with high EMS scores, because they appear to be less effective in regulating their responses on implicit measures of prejudice (Devine et al., 2002; Amodio et al., 2003). High EMS individuals are motivated by social (i.e., external) pressures to respond without prejudice. Hence, to be eligible for Stage II, participants needed to score above the sample median on both the IMS and EMS for both target social categories.

The third section of the survey included four *rating thermometers* (e.g., Herek, 2000) that required participants to give a single attitude rating (between 0° and 100°) for each of four different social groups: (a) Black individuals, (b) Jewish individuals, (c) homosexual individuals, and (d) fat individuals. A thermometer rating of 0° indicated an extremely unfavorable (cold) attitude toward a particular group, whereas a rating of 100° indicated an extremely favorable (warm) attitude toward that group. Here, we were looking for individuals who indicated a favorable rating (≥60°) toward all social groups.

#### Stage II: laboratory experiment

Participants who met the selection criteria described above were contacted telephonically and invited to attend the laboratory session. A white female experimenter tested each participant individually after informed consent had been obtained.

At the beginning of the session, participants were informed that the purpose of the study was the investigation of prejudiced attitudes among university students. They were told that psychology literature shows that there is a significant discrepancy between what people say they feel, and what they really feel, toward different social groups. Further, they were told that our hypothesis was that this implicit-explicit discrepancy has become smaller in recent years due to the educative value of the media and the contemporary political and cultural zeitgeist. Finally, they were told that they had been selected specifically for this study based on the results of the online survey (i.e., the survey had shown that they held positive explicit attitudes toward most social groups), and that we would now test their implicit attitudes toward these same social groups. The true nature of the experiment was thus withheld from participants because knowing that our aim was to elicit moral emotions might have invalidated their responses due to demand characteristics. The actual intention of the study, however, was revealed at the end of the session, during a thorough debriefing.

#### Questionnaires

After presentation of the cover story, participants completed several personality and mood questionnaires. These included the Positive and Negative Affect Schedule [PANAS; Watson et al., 1988; used to measure general levels of positive affect (PA) and negative affect (NA)], and the Affect Intensity Measure (AIM: Larsen and Diener, 1987; used to measure the intensity with which positive and negative emotions are typically experienced). Participants also completed the Behavioral Inhibition System and Behavioral Activation System scales (BIS/BAS; Carver and White, 1994) to measure their tendencies toward behavioral inhibition and behavioral activation sensitivity. Individuals with high BIS sensitivity should be naturally inclined to fixate on possible threats or punishment in their environment, and should be prone to experience negative affect (Gray, 1975, 1982; Gray and McNaughton, 2000). In contrast, individuals with high BAS sensitivity should be more responsive to cues of reward and more prone to experience positive affect.

We included the PANAS, AIM, and BIS/BAS so that we could investigate whether there were any meaningful relationships between affective traits and the self-reported magnitude of emotion elicited by our manipulation.

#### Emotion elicitation paradigm: modified IATs

After completing these questionnaires, participants were introduced to the IAT section of the protocol, which took approximately 25 min to complete. The researcher explained the task with the help of a detailed PowerPoint presentation, which was displayed on a 13″ computer monitor situated approximately 0.5 m from the participant. The presentation, which consisted of timed slides, also introduced the different social groups that participants would encounter during the IATs.

Prior to starting the study IATs, participants were required to do a practice IAT (on the topic of disability) on the Project Implicit website (https://implicit.harvard.edu/implicit/). This procedure helped to familiarize them with the task, and served to increase the credibility of our experimental paradigm. Before beginning the study IATs, participants were encouraged to approach the researcher if anything was unclear, and were also informed that the researcher would receive immediate feedback on their IAT results via a networked computer monitor on the other side of the laboratory.

We used a modified version of the conventional IAT (see Nosek et al., 2005 for more details) to elicit moral-negative and moral-positive affect in the selected low-prejudice individuals. In short, after the reaction-time task during which different concepts were paired with the words “good” or “bad,” our paradigm provided participants with pre-programmed response feedback (see Appendix). We used six different IATs, divided into three different response categories: Neutral, Positive, and Negative. The topics for the Neutral IATs were sports (preference for *swimmers* vs. *runners*) and facial hair (preference for *facial hair* vs. *no facial hair*). The topics for the Positive IATs were weight (preference for *fat* vs. *thin* people) and religion (preference for *Judaism* vs. *other religions*). The topics for the Negative IATs were sexuality (preference for *gay* vs. *straight* people) and race (preference for *Black* vs. *White* people).

Feedback for the Neutral IATs, unlike that of the Positive and Negative IATs, was not pre-programmed; instead, the results reflected the participant’s true implicit attitudes. The Neutral IATs were not expected to elicit any strong positive or negative affect, however, because these option categories were not controversial in a social sense, and hence any preference would only be a matter of personal choice.

The IATs crucial to the elicitation of moral-negative affect were the two in the Negative IAT condition. All participants received the same manipulated feedback, which suggested that the associations they made during the task were indicative of significant discriminatory attitudes against Black and homosexual individuals, despite the fact that their self-report during the screening and selection stage had suggested they were not prejudiced against these groups. We anticipated that the combination of the negative feedback and the participant’s awareness of her results being observed by the researcher would be effective in eliciting feelings of guilt (and, to a lesser degree, shame and embarrassment).

The IATs crucial to the elicitation of moral-positive affect were the two in the Positive IAT condition. Participants again received manipulated feedback which suggested that the associations they made during the task were indicative of egalitarian attitudes toward people of all different weights/religions. These associations, then, confirmed the self-reports they had made during the screening and selection stage. We anticipated that receiving feedback indicating that they respect people from various religions equally, and likewise show no prejudice against fat people, would induce positive emotions of pride and satisfaction in participants. At the same time, these IATs validated participants’ non-prejudiced beliefs and therefore enhanced the credibility of our paradigm.

The IATs were pre-programmed in a pseudo-random order with the only constraint that a Neutral condition IAT was always presented first. We devised five counter-balanced presentation orders so that every sixth participant received the same string of IATs.

#### Measurement of state affect during the elicitation paradigm

After each IAT, the participant received a 10-s feedback slide on her performance. This feedback was followed by a 20-s slide that gave more elaborate and detailed feedback about the attitudes revealed by the just-completed IAT (see Appendix for IAT feedback). This elaboration and detail was aimed at strengthening the emotional experience felt in response to the brief feedback. After viewing the 20-s slide, participants were required to complete an *emotion checklist*. This measure, which we designed to verify the elicitation of target emotional states in response to the IAT feedback, asked participants to rate the degree to which they experienced each of seven different emotions (guilt, shame, embarrassment, anxiety, fear, pride, and satisfaction). They gave their ratings along a five-point Likert-type scale, ranging from 1 (*not at all*) to 5 (*very much*).

#### Post-experimental interview

At the conclusion of the experimental manipulation, each participant was interviewed individually. During this interview, the participant was first asked to choose, from a list of eight options (the same as above, plus neutral), the primary emotion she had experienced following each IAT. Participants were then carefully probed for any other emotional experiences as well, i.e., whether they experienced a different emotion to those listed in the emotion checklist. If the participant chose an emotion other than the anticipated target emotion (e.g., if she had primarily experienced anger or disbelief following presentation of the race IAT), she was requested to explain why she had not experienced that target emotion. Finally, each participant was debriefed, informed of the true nature of the study, and asked not to discuss the experiment with fellow students.

### Statistical analysis

No inferential statistical analyses were performed on the Stage I measures; these served only as instruments to select participants suitable for Stage II.

The data of primary interest were those derived from the emotion checklists participants completed following each of the six IATs. We took two steps to reduce the number of dependent variables in the major inferential analyses. First, data from the checklists for IATs in the same response category were collapsed (e.g., the race and sexuality IAT emotion ratings were collapsed, and the weight and religion IAT emotion ratings were collapsed). Statistical analyses indicated that there were no significant affect differences between IATs in the same response category (*p*s > 0.06); our approach was thus justified. Second, we collapsed the data on each checklist into three emotion indices: (a) moral-negative emotion (consisting of the mean of ratings for guilt, shame, and embarrassment); (b) moral-positive emotion (mean of ratings for pride and satisfaction); and (c) basic-negative emotion (mean of ratings for fear and anxiety). Statistical analyses indicated that there were no significant differences between ratings of guilt, shame, and embarrassment in the Negative IAT condition (*p* > 0.60). However, participants’ ratings of satisfaction were significantly higher than that of pride in the Positive IAT condition (*p* < 0.01). Our decision to collapse pride and satisfaction was based on the notion that pride and moral self-satisfaction are typically felt in response to actions that are in accordance with one’s moral norms (Montada, 1993). Hence, each participant generated three emotion index scores (moral-negative, moral-positive, and basic-negative) for each of the Negative, Positive, and Neutral IAT sets.

Following these data manipulations, our statistical analyses proceeded across four stages. First, we conducted a 3 (IAT stimulus condition: Neutral, Positive, Negative) × 3 (emotion type: moral-negative, moral-positive, and basic-negative) repeated-measures ANOVA. This analysis explored whether the Negative and Positive IATs had elicited the target emotions most strongly (e.g., whether the Negative IATs had elicited moral-negative emotions more strongly than the Positive and Neutral IATs). In instances where the assumption of sphericity was violated, the degrees of freedom were corrected using Greenhouse–Geisser epsilon corrections. These correction factors are reported.

Second, we conducted two separate one-way repeated-measures ANOVAs on emotion index data from within IAT conditions. The first compared the moral-negative, moral-positive, and basic-negative data that followed the Positive IATs, and the other did the same comparison for the Negative IATs. These analyses sought to determine whether the specified target emotions had been most strongly elicited within the related IAT (e.g., whether the Negative IATs had elicited the moral-negative emotions more strongly than the basic-negative and moral-positive emotions).

Third, to investigate potential relationships between individual differences in affective traits and the elicitation of target emotions, we generated zero-order correlations between the data from the experimental questionnaires (PANAS, AIM, and BIS/BAS) and those from the emotion indices. Finally, we examined post-experimental interview data in an attempt to identify aspects of an individual’s personal characteristics and social/familial relationships that might have impacted the effective elicitation of target emotions, but that were not measured during Stage I. The Stage II sample was then refined further by excluding those individuals with potentially confounding personal characteristics and/or social/familial relationships, and the statistical analyses described above were repeated on this refined group.

## Results

### Stage I: Screening data

**Figure 1:**
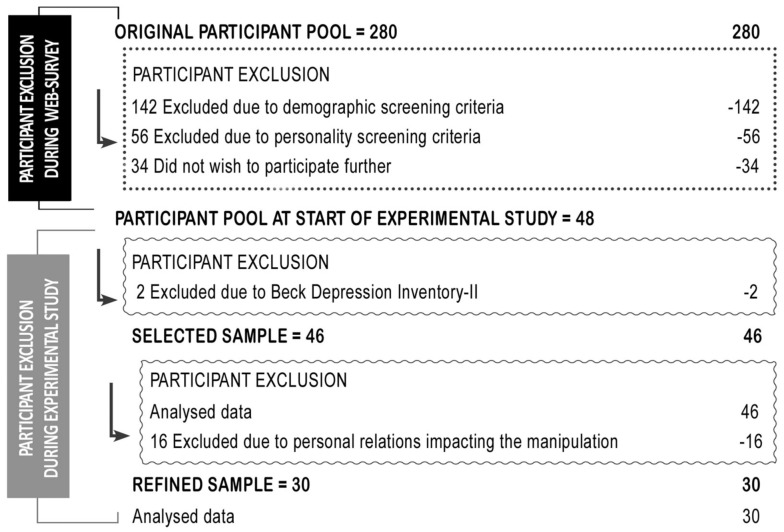
**Flowchart detailing the number of participants across the various stages of the study**.

Figure 1 demonstrates the flow of participants through the different stages of the study. As can be seen, more than 70% of the Stage I participants did not meet eligibility criteria for Stage II. Excluded individuals were those who scored below the IMS sample median (6.80) and below the EMS sample median (4.60) for both target categories, and/or rated their attitude toward one/more social group(s) as below 60° on the rating thermometers.

### Stage II: Self-reported emotions during the elicitation paradigm

Table 1 shows descriptive data from the self-report emotion checklists for each IAT.

**Table 1:** **Self-reported emotions from 1 (not at all) to 5 (very much) during the IAT elicitation paradigm (*N* = 46)**.

Emotions	IAT condition					
	Positive		Negative		Neutral	
	Weight	Religion	Race	Sexuality	Sport	Facial hair
Moral-negative	1.51 (0.79)	1.20 (0.44)	2.90 (1.22)	2.57 (1.22)	1.40 (0.59)	1.61 (0.87)
Guilt	1.57 (0.81)	1.24 (0.67)	2.87 (1.33)	2.50 (1.36)	1.50 (0.84)	1.52 (0.86)
Shame	1.52 (0.91)	1.22 (0.59)	2.87 (1.38)	2.54 (1.36)	1.28 (0.54)	1.50 (0.94)
Embarrassment	1.46 (0.66)	1.15 (0.56)	2.96 (1.43)	2.67 (1.42)	1.41 (0.75)	1.80 (1.07)
Basic-negative	1.26 (0.56)	1.29 (0.61)	1.76 (0.92)	1.47 (0.65)	1.61 (0.87)	1.36 (0.59)
Fear	1.11 (0.38)	1.15 (0.42)	1.54 (0.89)	1.24 (0.57)	1.30 (0.73)	1.15 (0.42)
Anxiety	1.41 (0.75)	1.43 (0.91)	1.98 (1.13)	1.70 (0.92)	1.91 (1.13)	1.57 (0.91)
Moral-positive	2.40 (1.32)	2.67 (1.35)	1.43 (0.79)	1.35 (0.71)	2.39 (1.05)	1.90 (1.11)
Pride	2.11 (1.32)	2.46 (1.57)	1.37 (0.83)	1.43 (0.89)	2.07 (1.18)	1.70 (1.19)
Satisfaction	2.70 (1.31)	2.89 (1.48)	1.50 (0.94)	1.26 (0.57)	2.72 (1.26)	2.11 (1.22)

*Data presented are means, with standard deviations in parentheses*.

#### Emotion elicitation across IAT conditions

**Figure 2:**
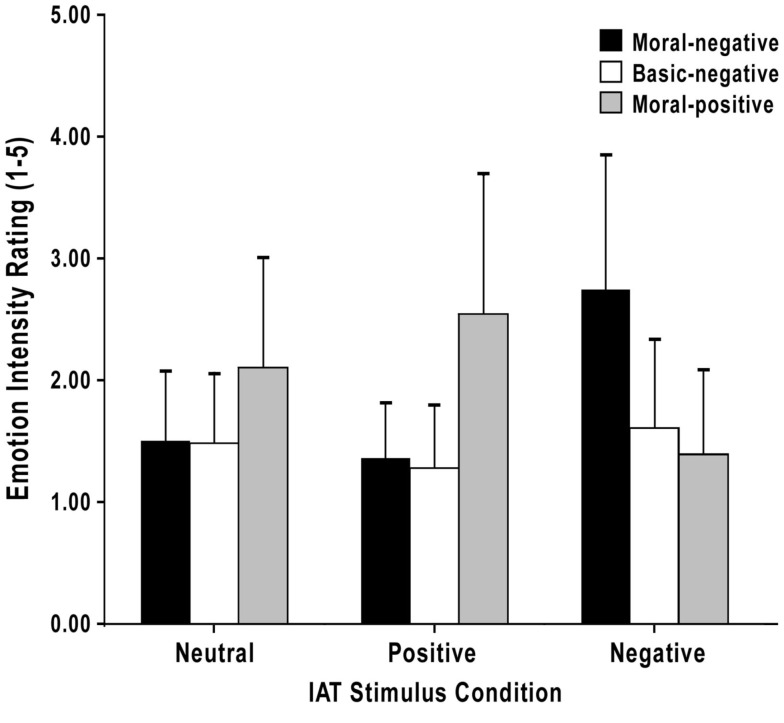
**Mean ratings for each emotion index following the Neutral, Positive, and Negative IATs**. Error bars indicate standard deviations.

Figure 2 shows that the Negative IATs (race and sexuality) elicited moral-negative emotions more strongly than the Positive IATs (weight and religion), whereas the opposite was true for the moral-positive emotions. Inferential statistical analyses supported these impressions: the two-way (stimulus condition × emotion type) repeated-measures ANOVA detected a significant main effect for stimulus condition, *F*(1.69, 75.91) = 8.74, *p* < 0.001, ω^2^ = 0.12, ε = 0.84, as well as for emotion type, *F*(1.60, 71.92) = 13.83, *p* < 0.001, ω^2^ = 0.28, ε = 0.80. Moreover, the interaction was significant, *F*(2.28, 102.37) = 50.56, *p* < 0.001, ω^2^ = 0.66, ε = 0.57; emotion ratings therefore varied depending on the stimulus condition.

To break down the interaction, we performed several *post-hoc* comparisons. Significant interactions were revealed when comparing the Negative IAT condition to the Neutral IAT condition in terms of scores on the moral-negative emotion index compared to scores on both the basic-negative emotion index (*p* < 0.001, *r* = 0.72) and moral-positive emotion index (*p* < 0.001, *r* = 0.80). These interactions reflect the fact that scores on the moral-negative emotion index increased significantly more than scores on the basic-negative and moral-positive emotion indices in the Negative IAT condition compared to the Neutral IAT condition.

Similarly, significant interactions were revealed when comparing the Positive IAT condition to the Neutral IAT condition in terms of scores on the moral-positive emotion index compared to scores on both the moral-negative emotion index (*p* = 0.01, *r* = 0.36) and basic-negative emotion index (*p* = 0.001, *r* = 0.48). Therefore, scores on the moral-positive emotion index increased significantly more than scores on the moral-negative and basic-negative emotion indices in the Positive IAT condition compared to the Neutral IAT condition.

Finally, when the Negative IAT condition was compared to the Positive IAT condition, scores on the moral-negative emotion index again increased significantly more than scores on both the basic-negative and moral-positive emotion indices (*p*s ≤ 0.001, *r*s > 0.70).

#### Emotion elicitation within IAT conditions

Figure 2 shows that, following each of the Positive IATs (weight and religion), participants reported experiencing moral-positive emotions more strongly than moral-negative and basic-negative emotions. A one-way repeated-measures ANOVA conducted on these data, and subsequent Bonferroni *post-hoc* tests, confirmed there was a significant main effect for emotion index, *F*(1.27, 57.20) = 39.73, *p* < 0.001, ω^2^ = 0.43, ε = 0.64, and that moral-positive emotions were elicited significantly more strongly following the Positive IATs than were the moral-negative and basic-negative emotions, *p*s < 0.001, *r*s > 0.68. There was no significant difference in the strength of elicitation of moral-negative and basic-negative emotions following the Positive IATs (*p* = 0.90).

Similarly, Figure 2 shows that, following each of the Negative IATs (race and sexuality), participants reported experiencing moral-negative emotions more strongly than moral-positive and basic-negative emotions. A one-way repeated-measures ANOVA conducted on these data, and subsequent Bonferroni *post-hoc* tests, confirmed there was a significant main effect for emotion index, *F*(1.52, 68.54) = 33.69, *p* < 0.001, ω^2^ = 0.40, ε = 0.76, and that moral-negative emotions were elicited significantly more strongly following the Negative IATs than were the moral-positive and basic-negative emotions, *p*s < 0.001, *r*s > 0.67. There was no significant difference in the strength of elicitation of moral-positive and basic-negative emotions following the Negative IATs (*p* = 0.37).

### Associations between personality measures and elicited emotions

Table 2 shows descriptive data for the Stage II questionnaires, as well as zero-order correlations between these questionnaire measures and target emotions obtained from the IAT emotion checklists. Participants’ scores for the PANAS and BIS/BAS scales were comparable to that of a random female sample (Watson et al., 1988; Carver and White, 1994), whereas their AIM scores were in the average range (Larsen, 1984). Consistent with previous work, participants’ BIS and BAS scores were also uncorrelated (*r* = −0.05, *p* = 0.72). Non-parametric tests were performed on data that did not meet assumptions underlying parametric testing. Because so many correlations were computed, we interpreted only those significant at the 1% level.

**Table 2:** **Correlations between questionnaire scores and self-reported affect in the positive and negative IAT conditions (*N* = 46)**.

	Mean (SD)	Negative IATs		Positive IATs
		MOR-NEG	BAS-NEG	MOR-POS
**SCREENING QUESTIONNAIRES**				
PA	33.96 (6.65)	–	–	0.08
NA	19.35 (5.76)	0.31*	0.21	–
AIM total	154.59 (16.10)	0.13	−0.01	−0.04
BIS	22.00 (3.54)	0.47**	0.27	0.20
BAS total	41.04 (4.92)	−0.12	−0.45**	−0.25
Drive	11.11 (2.15)	−0.19	−0.34*	−0.21
Fun seeking	12.57 (2.66)	−0.12	−0.31*	−0.02
Reward responsiveness	17.37 (1.69)	0.08	−0.30*	0.37*

*PA, positive affective schedule; NA, negative affective schedule; AIM, affect intensity measure; BIS, behavioral inhibition system; BAS, behavioral approach system; MOR-NEG, moral-negative emotions; BAS-NEG, basic-negative emotions; MOR-POS, moral-positive emotions*.

*p < 0.05 (two-tailed). **p < 0.01 (two-tailed).

Significant associations were those between BIS scores and moral-negative emotions following the Negative IATs. Further investigation of the relationship of the BIS and emotions elicited by the Negative IATs, through separation of the moral-negative index into its three constituent emotions (i.e., guilt, shame, and embarrassment), showed that there was also a significant positive correlation between BIS sensitivity and self- reported guilt as well as shame, *r*s = 0.52, *p*s < 0.001, but not embarrassment, *r* = 0.29, *p* = 0.06.

Significant negative correlations were also detected between BAS sensitivity (including BAS Total, as well as scores on the Drive, Fun seeking, and Reward responsiveness subscales) and the basic-negative emotions elicited by the Negative IATs.

### Personal characteristics: Potential impact on strength of elicitation

The post-experimental interviews were important to establish which emotions participants felt most strongly following each IAT. During these interviews we established that, following presentation of the Positive IATs, 34 participants (74% of the entire sample) experienced mostly satisfaction, whereas 4 (8.7%) felt mostly pride, and 7 (15.3%) felt neutral. In comparison, following presentation of the Negative IATs, 23 participants (50%) experienced mostly guilt, 2 (4.3%) felt mostly shame, and 5 (10.9%) felt mostly embarrassed. Sixteen participants, however, reported experiencing only disbelief, anger, or surprise in response to the Negative IAT feedback, which claimed that they held prejudiced attitudes toward Black and homosexual people.

Further questioning revealed that these 16 participants had personal characteristics and/or social-familial relationships that may have impacted on the success of the emotion elicitation paradigm (and, in particular, on the likelihood of eliciting guilt following the Negative IATs). Specifically, during the interviews we learned that three participants had a Black partner, two had a Black relative, and eight had a Black best friend. Clearly, these participants might not have believed the bogus feedback given to them following their completion of the race IAT, and so might not have experienced guilt in response to that manipulation. We also learned during the interviews that nine participants had close friends who were homosexual. Again, these facts might have made the emotion induction less plausible, thereby preventing the elicitation of specific feelings of guilt.

Hence, we re-ran the same analyses described above, this time excluding the data from those 16 participants identified as having potentially confounding personal characteristics and/or social-familial relationships. We expected that trends in the data and observed relationships would become more significant in this refined sample (*N* = 30).

**Figure 3:**
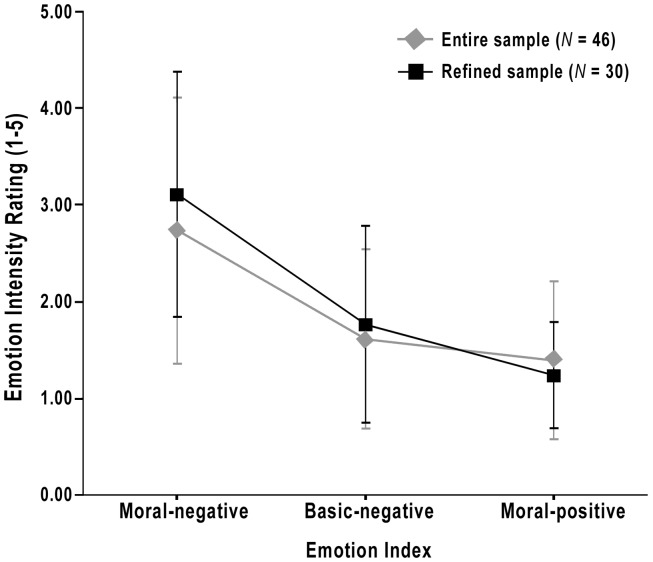
**Strength of elicited emotions following only the Negative IATs for the entire Stage II sample (*N* = 46), as well as for the refined sample (*N* = 30)**. Error bars indicate standard deviations.

These expectations were confirmed: as Figure 3 illustrates, in the refined sample, moral-negative emotions were elicited even more strongly by the Negative IATs (*M* = 3.11, *SD* = 0.93) than they were in the original sample (*M* = 2.74, *SD* = 1.10), compared to basic-negative and moral-positive emotions, *F*(2, 58) = 56.33, *p* < 0.001, ω^2^ = 0.63. In addition, the correlation between BIS sensitivity and the moral-negative emotions elicited by the Negative IATs increased from *r* = 0.47 (*p* < 0.01) to *r* = 0.55 (*p* < 0.01).

## Discussion

Developing methods to investigate moral emotions within a laboratory setting has been an ongoing challenge for researchers interested in learning how these emotions affect behavior and motivation (Tangney, 1996; Eisenberg, 2000; Lewis, 2000; Tracy and Robins, 2007). Perhaps the biggest confounding factor with existing elicitation methods is that the participant is not placed in emotion-evoking situations where he/she is the causal agent within a real situation, i.e., as the person who performed the embarrassing, shameful, or praiseworthy act. Rather, participants are often asked to *imagine* or *remember* an emotional event with themselves as the protagonist. The present study documents the development of a novel method to elicit moral emotions as salient affective states relevant to the participant. To this end, the paradigm made use of pre-programmed feedback of either high or non-existent prejudice on several IATs designed to elicit moral-negative and moral-positive emotions in low-prejudice individuals.

### Subjective emotion reports

Our paradigm, which involved a deceptive cover story and an IAT-based prejudice manipulation, proved effective: subjective emotion reports indicated that specific emotions of guilt, and to a lesser extent pride, were elicited. Specifically, our first set of analyses confirmed that moral-negative emotions (guilt, shame, and embarrassment) were elicited significantly more strongly by the Negative IATs than by the Positive and Neutral IATs. Similarly, moral-positive emotions (pride and satisfaction) were elicited significantly more strongly by the Positive IATs than by the Negative and Neutral IATs. Our second set of analyses compared the strength of emotional experience as it occurred within each of the Negative and Positive IAT conditions. These analyses showed that moral-positive emotions were elicited more strongly by the Positive IATs than were the moral-negative and basic-negative emotions (fear and anxiety). Similarly, moral-negative emotions were elicited more strongly by the Negative IATs than were the moral-positive and basic-negative emotions.

In sum, these two sets of results showed that the specific IATs elicited the targeted emotions effectively, but also that, when elicited, the target emotions were experienced significantly more strongly than the non-target emotions.

To determine whether individual target emotions of guilt and pride were elicited differentially by the emotion manipulation, we looked at data obtained during the post-experimental interviews. Participants were asked to indicate and explain which emotion they experienced most strongly after presentation of each IAT. Critically, the experimenter explained the meaning of certain emotions (e.g., shame and guilt) to avoid artifacts due to the erroneous labeling of emotions. These data indicated that, following the Negative IATs, participant affective experience was predominantly that of guilt. Apart from those individuals who were identified as having personal characteristics and/or social-familial relationships that prohibited them from believing the pre-programmed IAT feedback, participants furthermore reported that they found the IAT feedback convincing, and did not suspect that their IAT results were pre-programmed.

By comparison, post-experimental interview data indicated that, following the Positive IATs, participant affective experience was predominantly that of satisfaction (although participants reported significantly higher levels of pride during the emotion elicitation paradigm). Several authors have argued that we may feel pride when we uphold or act in accordance with our personal moral values (Haidt, 2001; Moll et al., 2005; Zahn et al., 2009). This response is also predicted by Duval’s objective self-awareness theory, which states that congruity between self and some personal standard should result in positive affect (Duval and Wicklund, 1972; Duval and Silvia, 2002). It is therefore unclear why the IAT paradigm did not succeed in producing strong pride.

A feasible explanation may be that participants experienced the Positive IAT feedback as merely cognitively pleasing/satisfying (Levenson, 2003), rather than something that made them feel proud about their behavior. Participants’ responses may also have been subject to social desirability concerns: low-prejudice individuals may regard it as socially unacceptable to feel proud of responding in a non-prejudiced fashion when that is how they believe one should respond anyway. A third explanation for our female participants’ low ratings of pride concerns possible gender-specific differences in emotional experience and expression. In this regard, evidence suggests that pride is an emotion expressed more frequently or intensely by males (Collins and Frankenhaeuser, 1989). Pride has become associated with maleness because it is thought to convey status to one’s social group, and males typically enjoy higher status than women in Western (and many non-Western) societies (Tracy and Robins, 2008). A final possibility is that participants no longer felt much pride after the emotion elicitation paradigm, given that positive events tend to be weaker and more short-lived than equally strong negative events (Baumeister et al., 2001; Larsen and Prizmic, 2004).

A common confounding factor in moral emotion elicitation studies is that the purity of the emotional state is often compromised because the paradigm may also evoke other emotions in parallel (Takahashi et al., 2004). The current IAT elicitation paradigm was designed to elicit specific feelings of guilt, based on findings from the literature on prejudice and self-discrepancy theory: this body of research suggests that low-prejudice individuals should experience specific feelings of guilt and remorse when they transgress their own internalized moral standards (Higgins, 1987; Plant and Devine, 1998). In the current study, however, the Negative IAT condition was marked by elevated self-ratings of several negative emotions, in particular shame and embarrassment, in addition to guilt. Shame and embarrassment were thus also felt, despite the fact that most participants reported (during the post-experimental interview) that guilt was their overriding feeling following the Negative IATs.

The subjective emotion reports following the Negative IATs may be explained by lay-people’s tendency to confuse distinctions between guilt and shame in everyday situations (Tangney and Dearing, 2002). Alternatively, elevated ratings of shame and embarrassment may have been stimulated by the experimental context. For example, Finger et al. (2006) reported elevated ratings of guilt, shame, and embarrassment in response to stories portraying victim-based moral transgressions. Their results, however, indicated that the presence of an audience triggered significantly higher ratings of embarrassment and shame in response to moral transgressions, but did not impact significantly on ratings of guilt. Ratings of embarrassment and shame in the present investigation may therefore have been augmented by a “public” factor, because participants were aware that their performances were being observed by the experimenter.

A final way to conceive of the subjective emotion reports obtained in the present study is to view shame and embarrassment as part of the emotional profile of guilt. It may well be that the case for “pure” guilt resulting from a social transgression is questionable, i.e., that some element of shame (or another negative emotion) may be present in most prototypical guilt responses. In moral transgression, for example, a person may feel guilty for violating a social standard while at the same time feeling embarrassed for being caught doing so, and feeling shameful about his/her own shortcomings (Eisenberg, 2000). In a similar vein, pride has also previously been conceptualized as the mean response to ratings of pride and satisfaction (Williams and DeSteno, 2008).

### Individual differences

Screening procedures are commonplace in emotion elicitation research, where it is of paramount importance that participants experience the specific emotions under investigation (e.g., Boiten, 1996; Reiman et al., 1997). Participants in the current study underwent a relatively stringent selection process, based on demographic and personality-based criteria applied during Stage I. Although this procedure may be associated with some limitations (discussed below), our results illustrated the value of employing such a set of criteria in identifying suitable candidates for inclusion in an emotion elicitation study of this nature. During the post-experimental interviews, for example, we discovered that some participants’ personal relations, such as having Black relatives or partners, impacted significantly on the success of the emotion manipulation. This finding was unexpected, given that one would expect such individuals’ motivations to respond without prejudice to be primarily internally motivated (i.e., high IMS/low EMS, unaffected by social desirability concerns), not internally *and* externally motivated, as specified by our selection criteria. A further adaptation of the selection process should therefore be to screen out individuals who have personal relations, such as close cross-racial friendships, which could prevent them from believing the manipulated IAT feedback and, thus, from experiencing guilt.

The value of implementing personality-based screening criteria was also demonstrated by the correlations observed between a number of Stage II questionnaire scores and self-reported emotion ratings. These correlations highlighted associations between certain personal characteristics and the degree of elicited affect. In particular, we found significant correlations between dispositional BIS and BAS sensitivities and subjective emotion responses: whereas BIS scores correlated positively with moral-negative emotions, BAS scores (including all BAS subscales) showed a strong inverse relationship with basic-negative emotions. These findings support the idea that individuals sensitive to aversive events or punishment cues (i.e., those with high BIS scores), would be likely to experience intense negative affect (in this case, guilt) in threatening situations (Gray, 1982). Conversely, individuals who score high on the BAS subscales may be insensitive to BIS warnings or cues of punishment, focusing their attention instead on cues of incentive (Patterson et al., 1987; Patterson and Newman, 1993). They may therefore be less likely, in general, to experience negative affect.

In summary, pre-experiment screening measures could be tailored to reflect each individual’s sociodemographic and personality characteristics, in order to optimize the elicitation of specific target emotions. The current results suggest that there would be value in adding the BIS/BAS scales to the set of measures administered during the participant selection stage.

### Limitations and conclusions

The current study successfully elicited specific moral emotions based on a carefully constructed prejudice paradigm. A number of methodological limitations should be noted, however. The effectiveness of our paradigm relied heavily on participants fulfilling a set of stipulated eligibility criteria, but such a stringent selection process may also be associated with some disadvantages. For example, screening a large pool of participants can be time-consuming and labor-intensive: in the current study, we screened 280 individuals to obtain a sufficiently large sample of participants (*N* = 48). Additionally, the use of stringent screening criteria may bias the study sample so that it becomes unrepresentative of the general population, which may in turn affect the generalizability of the resulting data.

Although a pre-screening process that delivers a relatively small pool of eligible participants might not be ideal under every research circumstance, we argue that such an approach may be justified in some cases. First, most existing moral emotion elicitation paradigms do not succeed in eliciting real-time moral affect; a fresh approach is therefore warranted. Second, our screening criteria were put in place to ensure the elicitation of target emotions in *every* participant; hence, the paradigm may be of value for more expensive or time-consuming laboratory-based studies of emotion. Third, the screening process we employed is relatively simple: it involves recruitment from the general population, the measures are straightforward to complete and widely available, the entire screening can be delivered via a survey website, and the choice of which candidates to include/exclude is easily performed by calculating sample descriptive statistics. Finally, we feel it is important to point out that the large number of individuals excluded from our study reflected both the multicultural nature of the South African population, as well as the significant amount of interpersonal prejudice still present in our country. This situation may be less extreme in other societies; the effectiveness of our paradigm in a more random sample is thus a question for future research.

Another potential limitation of the study is that we did not include males in our sample. As mentioned previously, we adopted this recruitment strategy to avoid gender-related emotion effects. It remains an open question, then, as to whether the elicitation paradigm described here would deliver effects of the same magnitude in a male sample. Similarly, the IAT prejudice paradigm in its current form was designed to elicit moral emotions in a homogenous sample of college students (white, heterosexual, non-Jewish), in order for the IAT feedback to appear plausible. Certain adaptations would thus be necessary before the paradigm could be applied to individuals of other races and sexual orientations.

Two further methodological limitations are that (i) we did not counterbalance the topics of the Positive and Negative IATs between participants, and (ii) we did not probe basic positive emotions. Whereas the first limitation may have helped eliminate possible confounding factors associated with specific IAT contents, the second limitation poses a more serious concern. Because we did not assess basic positive emotions, we cannot be certain that our paradigm elicited moral-positive emotions selectively in the Positive IAT condition. However, given that pride and satisfaction are often used as the only measured emotions to validate the elicitation of pride (see, e.g., Webster et al., 2003; Williams and DeSteno, 2008), we felt that our approach was consistent with relevant approaches adopted by previous research. Additionally, we probed participants during the post-experimental interviews for any other emotions they may have felt during the experimental procedure.

Finally, as noted above, the pride manipulation appeared to be less successful than the guilt manipulation. It is impossible to determine within the current study’s context, however, whether these lower levels of self-reported pride were a result of the emotion manipulation method, social desirability concerns, or sex-related differences in emotional responding. Future studies may attempt to unravel these threads. Also related to this matter is the possibility that participants’ reports of heightened guilt were influenced by social conformity. Individuals’ awareness of social conventions may well have led them to conclude that guilt was the most appropriate emotion to select in response to the negative IAT feedback. Although this possibility may be difficult to distinguish from the experience of true guilt, one way of teasing apart these effects could be to include a prosocial measure in the experiment. Given guilt’s well-established association with prosocial motivation (Tangney and Dearing, 2002), those participants who experienced true guilt may be more inclined to engage in reparatory behaviors (see, e.g., Amodio et al., 2007).

To conclude, in a novel departure from previously published studies, we tested a prejudice paradigm to elicit moral emotions in real-time. We confirmed, using a highly selected sample, that our paradigm was effective: as predicted, the manipulated IAT feedback elicited moral-negative emotions (primarily guilt) under one set of conditions, and moral-positive emotions (satisfaction and pride) under another set of conditions. Furthermore, high BIS-sensitive individuals experienced stronger moral-negative affect, which supports the idea that guilt functions as a punishment cue (Monteith, 1993); in contrast, high BAS-sensitive individuals experienced reduced negative affect in general. We suggest that this paradigm may contribute greatly to the investigation of moral emotions as a means to induce these cardinal sensitivities effortlessly within an experimental context.

## Conflict of Interest Statement

The authors declare that the research was conducted in the absence of any commercial or financial relationships that could be construed as a potential conflict of interest.

## Appendix

### Implicit association tests

#### Neutral IAT condition

##### Sports IAT (preference for swimmers vs. runners)

Picture stimuli: images of swimmers; images of runners

Words/Attributes: Joy, Love, Peace, Happy, Wonderful, Pleasure, Glorious, Laughter, Agony, Terrible, Horrible, Nasty, Evil, Awful, Failure, Hurt

Result: (10 s)

Your data suggest little to no automatic preference for swimmers vs. runners.

Elaborated feedback: (20 s)

Generally, people with little to no automatic preference for swimmers compared to runners:

» Adapt well to being around people engaging in either sport.
» Have no preference for watching one sport over the other on television.
» Are not intimidated by either runners or swimmers.
» Will at different times enjoy either swimming or running.
» Have had positive experiences with both types of sports.
» Are comfortable socializing with either runners or swimmers.

OR

Result: (10 s)

Your data suggest a slight automatic preference for swimmers compared to runners.

Elaborated feedback: (20 s)

Generally, people with a slight automatic preference for swimmers compared to runners:

» Prefer being around people engaged in swimming compared to running.
» Prefer watching swimming rather than running on television.
» Are slightly more intimidated by runners than swimmers.
» Most times will prefer swimming instead of running.
» Have had slightly more positive experiences with swimmers compared to runners.
» Are slightly more comfortable socializing with swimmers compared to runners.

OR

Result: (10 s)

Your data suggest a slight automatic preference for runners compared to swimmers.

Elaborated feedback: (20 s)

Generally, people with a slight automatic preference for runners compared to swimmers:

» Prefer being around people engaging in running compared to swimming.
» Prefer watching running rather than swimming on television.
» Are slightly more intimidated by swimmers than runners.
» Most times will prefer running instead of swimming.
» Have had slightly more positive experiences with runners compared to swimmers.
» Are slightly more comfortable socializing with runners compared to swimmers.

##### Facial hair IAT (preference for facial hair vs. no facial hair)

Picture stimuli: images of people with facial hair; images of people with no facial hair

Words/attributes: joy, love, peace, happy, wonderful, pleasure, glorious, laughter, agony, terrible, horrible, nasty, evil, awful, failure, hurt

Result: (10 s)

Your data suggest little to no automatic preference for people with no facial hair compared to people with facial hair.

Elaborated feedback: (20 s)

Generally, people with little to no automatic preference for people with no facial hair compared to people with facial hair:

» Do not find people with facial hair more attractive than people with no facial hair.
» Treat people with facial hair with the same respect as people with no facial hair.
» Believe that everybody has the freedom to express their identity.
» Value the internal qualities of an individual rather than their external qualities.
» Do not make friendship decisions based on the presence or absence of facial hair.
» Do not stereotype people with facial hair as being unhygienic.

OR

Result: (10 s)

Your data suggest a slight automatic preference for people with no facial hair compared to people with facial hair.

Elaborated feedback: (20 s)

Generally, people with a slight automatic preference for people with no facial hair compared to people with facial hair:

» Find people with no facial hair slightly more attractive than people with facial hair.
» Treat people with facial hair with slightly less respect than people with no facial hair.
» Believe that people should have to conform to conventional appearance ideals.
» Value the external qualities of an individual slightly more than their internal qualities.
» Sometimes make friendship decisions based on the presence or absence of facial hair.
» Sometimes stereotype people with facial hair as being unhygienic.

OR

Result: (10 s)

Your data suggest a slight automatic preference for people with facial hair compared to people with no facial hair.

Elaborated feedback: (20 s)

Generally, people with a slight automatic preference for people with facial hair compared to people with no facial hair:

» Find people with facial hair slightly more attractive than people with no facial hair.
» Treat people with no facial hair with slightly less respect than people with facial hair.
» Believe that people should not have to conform to conventional appearance ideals.
» Value the internal qualities of an individual slightly more than their external qualities.
» Sometimes make friendship decisions based on the presence or absence of facial hair.
» Do not stereotype people with facial hair as being unhygienic.

#### Positive IAT condition

##### Weight IAT (preference for thin vs. fat people)

Picture stimuli: images of fat people; images of thin people

Words/Attributes: Nice, Healthy, Pretty, Happy, Wonderful, Pleasure, Successful, Beautiful, Disgusting, Unacceptable, Terrible, Horrible, Nasty, Awful, Failure, Unhealthy

Result: (10 s)

Your data suggest little to no automatic preference for thin vs. fat people.

Elaborated feedback: (20 s)

Generally, people with little to no automatic preference for thin compared to fat people:

» Attach value to eating healthily and exercising.
» Treat fat people with the same respect as anybody else.
» Do not believe that body weight determines success.
» Value the internal qualities of an individual rather than their external qualities.
» Do not make friendship decisions based on body weight.
» Do not stereotype fat people as being lazy.

##### Religion IAT (preference for Judaism vs. other religions)

Picture stimuli: Judaic images; Images of other religions (Buddhism, Christianity, Hinduism, Islam)

Words/Attributes: Celebrate, Happy, Friendly, Joyful, Laughter, Beautiful, Wonderful, Cheerful, Horrible, Angry, Awful, Unpleasant, Hate, Destroy, Brutal, Nasty

Result: (10 s)

Your data suggest little to no automatic preference for other religions compared to Judaism.

Elaborated feedback: (20 s)

Generally, people with little to no automatic preference for other religions compared to Judaism:

» Have no disregard for Jewish individuals.
» Believe that all religions are equal and should be treated with respect.
» Believe that every person should be allowed freedom to practice his/her own religion.
» Often believe strongly in human rights.
» Do not judge people based on their religious practices.
» Are themselves spiritual in some way.

#### Negative IAT condition

##### Race IAT (preference for Black vs. White people)

Picture stimuli: faces of Black people; faces of White people

Words/Attributes: Joy, Love, Peace, Wonderful, Pleasure, Glorious, Laughter, Happy, Agony, Terrible, Horrible, Nasty, Evil, Awful, Failure, Hurt

Result: (10 s)

Your data suggest a strong automatic preference for white vs. black people.

Elaborated feedback: (20 s)

Generally, people with a strong automatic preference for White compared to Black people:

» Are more afraid of Black people than White people.
» Often find themselves in racially skewed working environments.
» Often react uncomfortably upon meeting a Black person (e.g., by avoiding eye contact).
» Will avoid situations involving large crowds of Black people.
» Will never openly disregard black people, but always prefer white company.
» Would never have a romantic relationship with a Black person.

##### Sexuality IAT (preference for gay vs. straight people)

Picture stimuli: images/symbols related to gay people; images/symbols related to straight people

Words/Attributes: Joy, Love, Peace, Happy, Wonderful, Pleasure, Acceptable, Glorious, Unnatural, Horrible, Nasty, Awful, Failure, Offensive, Wrong, Disgusting

Result: (10 s)

Your data suggest a strong automatic preference for straight compared to gay people.

Elaborated feedback: (20 s)

Generally, people with a strong automatic preference for straight compared to gay people:

» Will never openly admit to judging gay people, but donot believe in gay rights.
» Do not have any close friends who are gay.
» Have stereotyped opinions about what gay people do and who they are.
» Avoid being associated with gay people.
» Believe that homosexuality is unnatural.
» Would prefer if homosexual marriages were illegal.
